# Introgression—Friend or Foe?

**DOI:** 10.1111/mec.17810

**Published:** 2025-05-28

**Authors:** M. Hindrikson, E. Tammeleht

**Affiliations:** ^1^ Department of Zoology University of Tartu Tartu Estonia

**Keywords:** admixture, *Canis lupus*, hybridization, Iberian wolf population, introgression

1

In this issue of Molecular Ecology, Sarabia et al. (2025), findings on potential adaptive introgression from dogs (Canis familiaris) into Iberian grey wolves (Canis lupus) present several fascinating and intriguing aspects. The study highlights a rare case of adaptive introgression, where genes from domestic dogs that were incorporated into the genome of Iberian grey wolves may have provided beneficial effects rather than deleterious ones. This challenges the common assumption that hybridization between wild species and domestic relatives primarily leads to negative outcomes. Six genes linked to immune response mechanisms and brain functions were found to carry alleles introgressed from dogs under positive selection in wild Iberian wolves, suggesting that the introgressed genes may help explain unique behavioural phenotypes observed in Iberian wolves (Figure 1), particularly their reduced dispersal compared to other wolf populations in Europe. Despite evidence for ancient gene flow between dogs and wolves over thousands of years, current study reports minimal recent hybridization events among contemporary Iberian wolf population (averaging around 0.6% recent dog ancestry). Understanding how adaptive traits could be introduced through introgression is vital for conservation efforts aimed at preserving genetic diversity within endangered species populations like the Iberian wolf.

Natural hybridization in the form of the interbreeding of two distinct taxa is a positive evolutionary force for introducing beneficial adaptive genetic variation into the genomic structure of species or populations Figure 1. The remarkable influence of hybridization and gene flow among canids in shaping phylogenetic relationships and population structures (Figure 2) underscores the potential significance of wolf‐dog hybridization on existing grey wolf populations, particularly as wolves, being one of the most studied carnivore species with an abundance of ancient and modern genomes available, provide an excellent model for examining the effects of hybridization and introgression. Indeed, in Sarabia et al. (2025) recent availability of entire genomes of both wolves and domestic dogs has been put to use for the benefit of the current Iberian wolf population: 150 whole genomes of Iberian and other Eurasian grey wolves as well as dogs originating from across Europe were analysed to assess the extent and impact of dog introgression.

**FIGURE 1:**
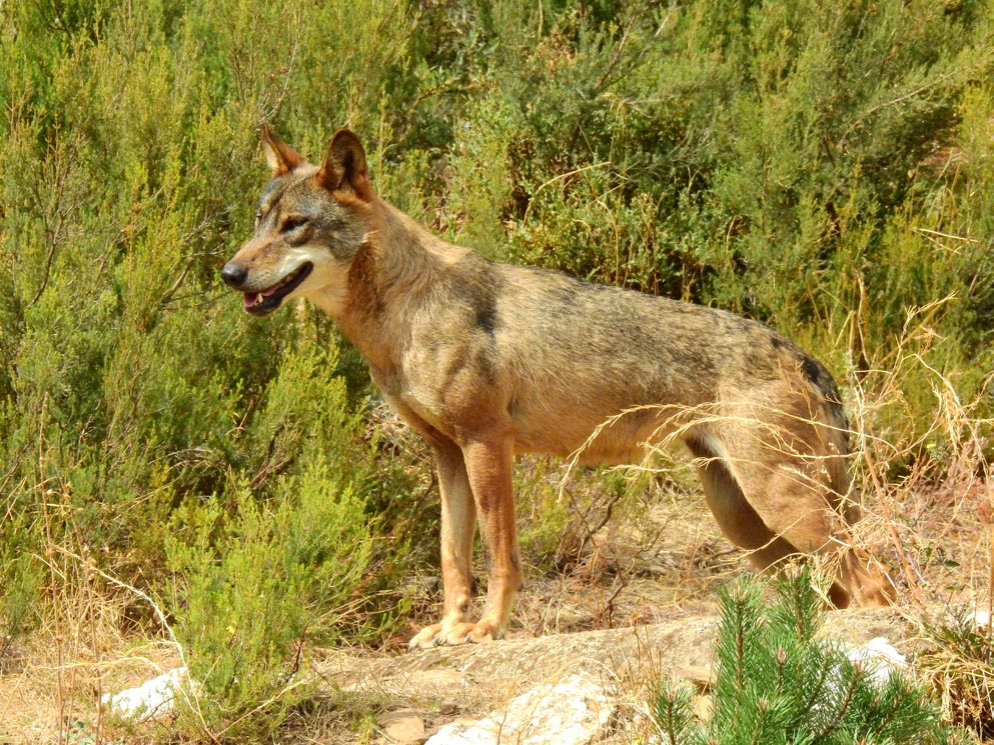
Iberian wolf individual. Iberian wolf with summer coat in north‐western Spain. Photo credit: Isabel Salado.

**FIGURE 2:**
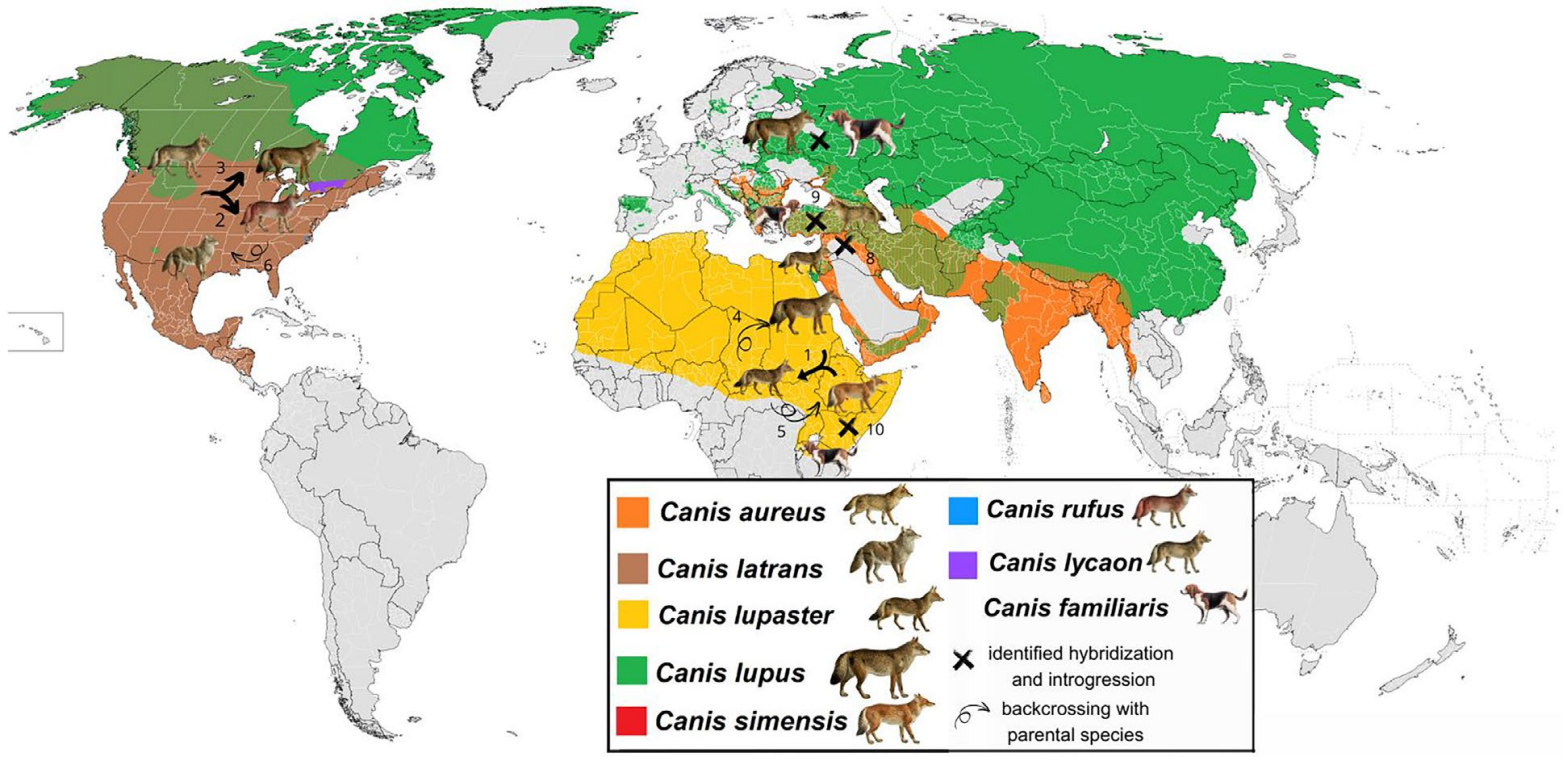
Map of interspecific hybridization. Interspecific hybridization in genus Canis has resulted not only in appearances of new species, such as African golden wolf ( *Canis lupaster* , 1) or indigenous forest wolves in America, which roamed the eastern forests of southern Canada to Florida and west to the Great Plains, now known as red wolf ( *Canis rufus* , 2) in the south and eastern wolf (*Canis lycaon*, 3) in the north, sharing a common evolutionary history of hybridization between wolves and coyotes ( *Canis latrans* ), but also has an impact on population structures of the existing ones (African golden wolf, after emerging as a separate species has been hybridising with both parental species populations—grey wolves ( *Canis lupus* , 4) and Ethiopian wolves ( *Canis simensis* , 5) and the same has happened with red wolf, who have been interbreeding naturally with coyotes (6) for at least a century, particularly in a specific area in eastern Texas, before going to extinct in 1980s. The grey wolves in the Great Lakes region are known to have a history of admixture with both coyotes and eastern wolves (Gopalakrishnan et al. 2018; Holdt et al. 2022). Hybridization and introgression between wolves and dogs has been identified in almost every wolf population in Europe (7). Furthermore, introgression between golden jackal and African wolves (8) and domestic dogs (9) has been shown in the Levant region (Martins et al. 2024). Ethiopian wolves have been shown to hybridise with domestic dogs (10) (Gottelli et al. 1994). Map and distribution of canid species: https://commons.wikimedia.orG/wiki/File:Canis_distribution.png (modified by the authors).

Hybridization between domesticated forms and their wild ancestors—anthropogenic hybridization—is mostly considered a threat to biodiversity, for example due to waste of reproductive effort of wild parental taxa. Wolf and dog, like all the other species in genus *Canis*, can interbreed and their fertile hybrids can backcross with both parental populations, leading to the introgression of domesticated genes into the wolf gene pool (Vilà and Wayne 1999). Hybridization between wolves and dogs has been a recurrent phenomenon since the domestication of dogs (Pilot et al. 2021), but it may be occurring at an accelerating rate in human‐dominated landscapes, such as Europe. Large‐scale surveys have confirmed ongoing and recent hybridization in several Eurasian wolf populations (e.g., Pilot et al. 2018, 2021; Salvatori et al. 2020).

Recently, Santostasi et al. (2025) applied an individual‐based model (IBM) to simulate the life cycle of wolves by projecting hybridization dynamics in a local wolf population and showed that lack of management led to complete admixture and genomic extinction. However, this may be true only in highly fragmented small wolf populations. For example, there seems to be no evidence of hybridization or introgression in the Scandinavian wolf population (Smeds et al. 2020). Despite finding small blocks of dog ancestry in the genomes of 62% wolves sampled from all Eurasian populations analysed, the wolf populations have maintained a distinct genetic profile from dogs, suggesting that hybridization and backcrossing have occurred at a low frequency (Pilot et al. 2018). By analysing 150 whole genomes from both Iberian and other Eurasian grey wolves, as well as dog populations across Europe and western Siberia, Sarabia et al. (2025) identified only about 5% ancient dog ancestry within the Iberian wolf population.

Introgression from domesticated species is generally believed to have predominantly negative impacts on wild populations, as genetic and phenotypic variations from domestication can be harmful to wild integrity, and the numerical dominance of domestic animals over their wild counterparts facilitates genetic swamping. The hybridization between wolves and dogs generally poses a conservation concern due to its potentially harmful long‐term evolutionary effects, including the loss of adaptive gene combinations, unique genotypes with distinct evolutionary histories, and a reduction in fitness and adaptive potential due to the introduction of maladaptive traits (Smith et al. 2022). However, the significant advancements in genetic tools over the past decades have enabled more detailed analyses and a deeper understanding of hybridization, introgression, and the genomic mechanisms underlying these processes. Sarabia et al. (2025) found some immune system gene variants from domestic animals to enhance the fitness of their wild relatives, besides genes influencing behavioural traits. Behavioural differences, such as decreased dispersal compared to other wolf populations, have previously been described in Iberian wolves. Moreover, examples from North American wolf populations show that one introgressed dog allele can increase individuals' fitness by conferring resistance to the canine distemper virus (Cubaynes et al. 2022). Miao et al. (2017) results illustrate the opposite direction of positive introgression: the introgression of hypoxia adaptive genes in wolves from the highland played an important role for dogs living in hypoxic environments, indicating that domestic animals could acquire local adaptation quickly by secondary contact with their wild relatives.

As a conclusion, there is a new wave of evidence surfacing from the genomes of wolves that introgression may not be so frequent as previously thought and moreover, hybridization in itself may provide wolf populations a way of acquiring new adaptations to a rapidly changing environment. European legislation requires that wolf–dog hybridization is mitigated through effective management, mostly meaning killing the hybrid animals. However, Sarabia et al. (2025) study nicely underpins that eliminating individuals possessing dog‐derived traits may prevent adaptations from being established in wolf populations and therefore we should, especially in Europe, where most wolf populations are presently large and expanding their range boundaries, let hybridization and furthermore, introgression, work their way.

## Author Contributions

The authors take full responsibility for his article.

## Conflicts of Interest

The authors declare no conflicts of interest.

## Data Availability

The authors have nothing to report.
